# Role of cAMP in Double Switch of Glucagon Secretion

**DOI:** 10.3390/cells10040896

**Published:** 2021-04-14

**Authors:** Jan Zmazek, Vladimir Grubelnik, Rene Markovič, Marko Marhl

**Affiliations:** 1 Faculty of Natural Sciences and Mathematics, University of Maribor, 2000 Maribor, Slovenia; jan.zmazek@um.si (J.Z.); rene.markovic@um.si (R.M.); 2 Faculty of Electrical Engineering and Computer Science, University of Maribor, 2000 Maribor, Slovenia; vlado.grubelnik@um.si; 3 Faculty of Education, University of Maribor, 2000 Maribor, Slovenia; 4 Faculty of Medicine, University of Maribor, 2000 Maribor, Slovenia

**Keywords:** pancreatic alpha cell, glucagon, cAMP, mathematical model, diabetes, cellular bioenergetics

## Abstract

Glucose metabolism plays a crucial role in modulating glucagon secretion in pancreatic alpha cells. However, the downstream effects of glucose metabolism and the activated signaling pathways influencing glucagon granule exocytosis are still obscure. We developed a computational alpha cell model, implementing metabolic pathways of glucose and free fatty acids (FFA) catabolism and an intrinsically activated cAMP signaling pathway. According to the model predictions, increased catabolic activity is able to suppress the cAMP signaling pathway, reducing exocytosis in a Ca^2+^-dependent and Ca^2+^ independent manner. The effect is synergistic to the pathway involving ATP-dependent closure of K_ATP_ channels and consequent reduction of Ca^2+^. We analyze the contribution of each pathway to glucagon secretion and show that both play decisive roles, providing a kind of “secure double switch”. The cAMP-driven signaling switch plays a dominant role, while the ATP-driven metabolic switch is less favored. The ratio is approximately 60:40, according to the most recent experimental evidence.

## 1. Introduction

Type 2 diabetes mellitus (T2DM) represents a significant public health problem, with more than 7% of the adult population being affected [1]. It is generally characterized by improper glucose homeostasis, mainly controlled by two hormones, insulin secreted from pancreatic beta cells and glucagon secreted from pancreatic alpha cells. While the insulin-secreting pancreatic beta cells and their physiological function have been thoroughly investigated over the past decades, the research focus has been shifting toward the glucagon [2] and the significance of alpha cell’s pathophysiology in T2DM. Moreover, while the consensus model for glucose-induced insulin secretion has been established [3], the model for glucose-induced signaling in alpha cells is still very much under debate. It is evident from the consensus model of insulin secretion that metabolic control is the crucial mechanism for glucose sensing and consequent insulin exocytosis. On the other hand, it seems that the alpha cell’s glucagon secretion mechanisms are much more complex, involving various pathways such as paracrine and juxtacrine signaling, electrical excitability, and intracellular signaling.

Although there exist substantial implications for paracrine regulation of glucagon secretion primarily due to the spatial distribution of alpha cells and vascular organization within the islet [4,5], it is now clear that glucose also inhibits glucagon secretion by a direct effect on alpha cells [6]. The importance of intrinsic regulation is particularly illustrated by the suppression of glucose-regulated glucagon secretion via the ablation of glucokinase [7], which is crucial for the establishment of glucose-dependent glycolytic flux. However, since glucose increase does not significantly reduce Ca^2+^ under hyperglycemia [8], other factors probably regulate glucagon exocytosis in alpha cells apart from depolarization-induced inactivation voltage-dependent calcium channels (VDCC). Such factors may include AMP-activated protein kinase (AMPK) signaling [9], cAMP-PKA/Epac [10], and other signaling pathways [11]. Due to the ability of cAMP to firmly amplify glucagon exocytosis in response to adrenaline [12] and inhibit glucagon secretion in response to the GLP-1 [13], the cAMP pathway has been of particular research interest [14]. Moreover, the cAMP concentration lowers with increasing glucose levels [10], indicating the additional intrinsic mechanism of cAMP formation.

In response to incretins or adrenaline, cAMP is generated by the transmembrane adenylyl cyclases (tmACs), while the intrinsic increase of cAMP in response to glucose is due to stimulation of soluble adenylyl cyclases (sACs) [15]. Unlike its transmembrane counterpart, the activity of which is modulated by the G-protein coupled receptors (GPCR), the sAC’s activity is regulated by bicarbonate (HCO_3_^−^), Ca^2+^ ions, and also influenced by ATP [16]. sAC catalyzes the generation of cAMP from ATP. The reaction rate obeys the Michaelis–Menten kinetics, determined by the *V*_max_ (the maximum rate of the reaction at the substrate saturation) and *K*_m_ (substrate concentration at which the reaction rate is half of *V*_max_). HCO_3_^−^ stimulation of sAC is consistent with the increase of *V*_max_ of its activity, while Ca^2+^ stimulates sAC by reducing its apparent *K*_m_ [17]. Mammalian sAC structure more closely resembles cyanobacterial ACs than tmACs, suggesting that the sAC acts as an evolutionarily conserved bicarbonate sensor [18]. The discovery led to a general concept of the intracellular metabolic sensor, presented by several papers [16,18,19,20,21], by which HCO_3_^−^ modulates the sAC activity in response to alterations in metabolic rate.

Intracellularly, HCO_3_^−^ is produced by dissociation of carbonic acid, which is generated when CO_2_ reacts with water. The reaction is catalyzed by the enzyme carbonic anhydrase (CA), allowing for the establishment of equilibrium between CO_2_ and HCO_3_^−^. Therefore, while the CO_2_ is generally considered a waste product of the oxidative metabolism, the perturbations in its concentration could implicitly influence the cAMP production via stimulation of sAC by HCO_3_^−^. Indeed, the colocalization of CA Ⅰ and glucagon has been found in alpha cells [22], which hints at its physiological role. With CA Ⅰ present, the reaction of carbonic acid formation and its dissociation to HCO_3_^−^ and H^+^ is in rapid equilibrium. Since the physiological range of HCO_3_^−^ concentrations are well below the enzyme’s saturation value, the changes in HCO_3_^−^ affect the sAC reaction velocity. Accordingly, perturbations of HCO_3_^−^ concentration coincide with the changes in cAMP concentration. Since CO_2_, HCO_3_^−^ and H^+^ concentrations are coupled according to the Henderson–Hasselbalch equation, the cAMP signaling pathway, which is defined by the sAC activity, responds to changes in the rate of intracellular metabolism (via mitochondrial CO_2_ production), pH (via glycolytic lactic acid production), and ionic fluxes [19]. The vital aspect of this cAMP-generating mechanism is that it is completely independent of the well-established ATP-sensing pathway. Recent experimental findings confirm that cAMP is directly controlled by the glucose independently of paracrine influences and that glucose prevents inhibition of glucagon secretion by fixing cAMP at high levels [23].

In addition to cAMP signaling, alpha cells possess another mechanism for regulating glucose-induced glucagon secretion, operating on the basis of the metabolism-induced increase in ATP concentration. Alpha cells are electrically excitable and equipped with the same type of ATP-sensitive potassium (K_ATP_) channels as beta cells [24,25]. The lowered conductance of K_ATP_ channels in alpha cells reduces electrical activity and glucagon exocytosis via depolarization-induced inactivation of ion channels involved in action potential firing and secretion [26]. Increased ATP concentration acts via depolarization-induced inactivation of the voltage-dependent calcium channels (VDCC). Especially crucial for glucagon exocytosis seem to be the P/Q-type VDCC [27], even though the L-type VDCCs mediate the majority of calcium influx under hypoglycemic conditions [26]. According to the model by [25], alpha cells generate large-amplitude Na^+^ currents under hypoglycemic conditions, which triggers Ca^2+^ entry through P/Q-type VDCC. Elevation of glucose produces a reduction in action potential height (but increase in frequency), which results in reduced Ca^2+^ entry through P/Q-type channels and, in turn, diminishes glucagon exocytosis. Calcium concentration correlates with glucagon exocytosis during hypoglycemia but uncouples during supraphysiological glucose levels [14,28]. With high glucose concentrations, elevated global calcium concentration prevails over the calcium elevations in microdomains near P/Q channels. On the other hand, elevated concentration of cAMP (achieved, e.g., by adrenaline or forskolin) produces an entirely different pattern, by which the increased Ca^2+^ current and exocytotic response result from an enhancement of the L-type component [12].

Intrinsically, glucose sensing is achieved by the establishment of the glucose-dependent glycolytic flux. Although under the same extracellular glucose concentrations, the alpha cell’s glycolytic flux is comparable to that in the beta cell [29], the ATP concentration, and therefore the energy state in the alpha cell is relatively steady across a wide range of glucose concentrations [30]. Data indicate that glucose fate is mainly anaerobic in alpha cells since the expression of lactate dehydrogenase (LDH) to FAD-linked glycerol-3-phosphate dehydrogenase GPDH is four-fold higher than in beta cells [31]. Consequently, the glucose oxidative capacity of alpha cells is only 1/3 to 1/6 of that in beta cells [31]. Apart from glucose, other metabolites such as amino acids and free fatty acids (FFA) contribute to the increase of hormone secretion. However, it has been shown that exposure to FFA is particularly essential [32] as the beta-oxidation of FFA significantly contributes to the increase in the cell’s energy state. It has been demonstrated that a higher load of FFA increases glucagon secretion in alpha cells [33]. Furthermore, the contribution of FFA oxidation was recently demonstrated by the pharmacological blockade of CPT1, which reduced glucagon secretion by approximately 40% [34]. The oxidation of glucose and FFA molecules produces a flux of other products, primarily CO_2_ and lactic acid, which is particularly significant in highly anaerobic alpha cells.

Experimental research on pancreatic alpha cells has been gaining attention in the past years, and several computational models reproducing the effects of elevated glucose concentration on glucagon exocytosis have been developed [35,36,37,38]. Computational models primarily focus on the electrophysiological changes, responding to the closure of K_ATP_ channels due to the effect of glucose on the increased alpha cell’s energy state. However, several recent articles have shown the crucial role of the cAMP signaling pathway in alpha cells [10,14,23,39], also without the presence of extracellular paracrine factors [40]. In this paper, we present a computational model incorporating vital metabolic and signaling pathways in alpha cells, leading to the switching on/off the glucagon secretion in alpha cells. Strict separation of the metabolic and signaling pathways enables quantitative assessment of their relative contributions. The metabolic pathways, which include the catabolism of glucose and FFA, primarily result in changes in ATP concentration, which impact the electrophysiology of the cell. In parallel, catabolism of nutrients also triggers the cAMP signaling pathway, which is caused by the metabolic outputs of CO_2_ and lactic acid. The model predictions show that both metabolic and signaling pathways contribute to a fine-regulated double switch for glucagon secretion. The contribution of the cAMP signaling pathway is prevalent compared to the ATP-driven cascade, operated via closing the K_ATP_ channels and the consequent direct effect of Ca^2+^ on the secretory machinery of glucagon secretion. The results are discussed in the context of the most recent experimental data [23].

## 2. Computational Model

**Figure 1:**
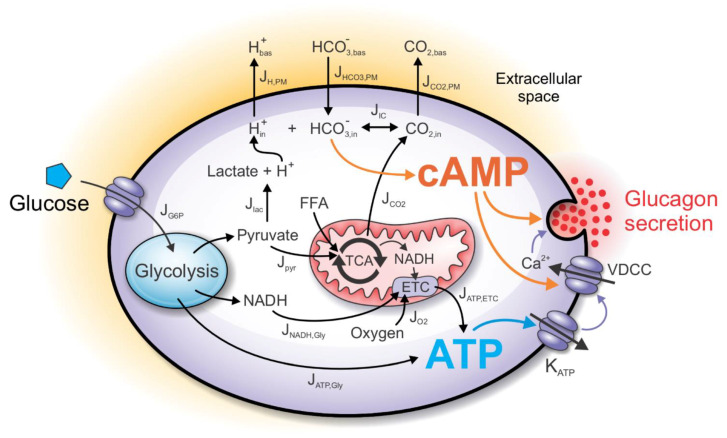
Schematic presentation of the computational model. Glucose input into alpha cells indirectly affects metabolic and signaling pathways, finally leading to the modulation of glucagon secretion. The metabolic component of the model includes catabolic processes of glucose and free fatty acids (FFAs), necessary for ATP generation. ATP is produced anaerobically during glycolysis, while the mitochondrial generation of ATP requires the influx of oxygen. In parallel to the metabolic component, the signaling component of the model is initiated by the glycolysis-derived lactic acid and mitochondria-derived CO_2,_ which together cause a shift of the bicarbonate buffer equilibrium in the intracellular space. Consequently, the intracellular bicarbonate concentration influences the intracellular cAMP concentration. ATP and cAMP together modulate glucagon secretion either by influencing calcium entry through voltage-dependent calcium channels (VDCC) or acting directly on the glucagon granule exocytosis.

The computational model incorporates the main intrinsic mechanisms by which alpha cells modulate glucagon secretion upon glucose stimulation (Figure 1). The glucose, entering the cell in a concentration-dependent matter, establishes a glycolytic flux, which increases ATP concentration. The cell’s energy state is linked to the glucagon secretion by the K_ATP_ channels, modulating the VDCC. The decreased calcium influx lowers calcium-dependent exocytosis of the glucagon granules. Moreover, the increased CO_2_ and lactate outputs influence cAMP production, which synergistically with increased ATP concentration regulates glucagon exocytosis both indirectly (by modulating Ca^2+^ influx) and directly (by modulating exocytotic machinery). The only input parameter of the model is plasma glucose concentration (see Equation (1)), while the alpha cell’s net rate of oxygen consumption is modeled based on the available experimental findings. Other metabolite concentrations shown in Figure 1 result from the computational analysis. We describe separately the metabolic and the signaling processes that contribute to glucagon secretion.

### 2.1. The ATP-Producing Metabolic Component

The computational model’s metabolic component incorporates vital metabolic processes (glycolysis, TCA cycle, beta-oxidation of FFA, and the electron transport chain) by which alpha cells aerobically and anaerobically produce ATP. The crucial factor for the glucose-induced increase in ATP concentration and the resulting inhibition of glucagon secretion is the anaerobic ATP production. It was previously shown that the reduction of glycolysis-produced ATP molecules significantly abolishes hormone exocytosis [38].

The main input to the model is the extracellular glucose concentration ([G]). After being transported into the cell, glucose enters the glycolytic pathway. The first step of the glycolysis is the conversion of glucose to glucose-6-phosphate (G6P), mediated by the low-affinity high-Km enzyme glucokinase (hexokinase Ⅳ), which is treated as the limiting step of glycolysis. Its dynamics in the alpha cell is modeled by:

(1) $$\begin{array}{c} J_{G6P}=J_{\max}\frac{{\left[G\right]}^{2}}{K_{m}^{2}+{\left[G\right]}^{2}}\; \end{array}$$

where *J*_G6P_ is the G6P production rate, *J*_max_ is the maximal reaction rate, and *K*_m_ is the glucose concentration at which *J*_G6P_ reaches half of the maximal value. Parameters *J*_max_ and *K*_m_ are chosen based on the experimental data for glucose concentrations G at 1 and 10 mM [31]. To match *J*_G6P_ glucose-dependent dynamics with experimental data, reported by Schuit et al., we used *J*_max_ = 7.2 μM s^−1^ and *K*_m_ = 5 mM. The dynamics of *J*_G6P_ (Equation (1)) were for the same set of parameters matched with experimental data in the previous work [38].

Glycolysis consists of 10 reactions, converting each 6-carbon glucose into two 3-carbon pyruvate molecules. The net yield of the investment and payoff phases of glycolysis are two molecules of anaerobically-produced ATP (*J*_ATP,gly_) per one glucose molecule entering the glycolytic pathway, which is given by:

(2) $$\begin{array}{c} J_{\mathrm{ATP},\mathrm{gly}}=2J_{G6P}\; \end{array}$$

Moreover, the payoff phase also yields two NADH and two pyruvate molecules per one glucose molecule. However, a fraction of the pyruvate (*p*_L_) is reduced to lactate in a redox reaction, consuming NADH as an electron donor. The proportion of pyruvate-to-lactate conversion is primarily dependent on the cell type. In the model by Wilson et al. [41], the *p*_L_ value is set to 20%. However, compared to beta cells, the value of *p*_L_ is notably higher in alpha cells due to relatively much higher expression of LDH [31]. We set the *p*_L_ = 0.9, matching the value of the previously published model [38]. Taken together, the rate of glycolytic NADH (*J*_NADH,gly_) and pyruvate production (*J*_pyr_) is given by:

(3) $$\begin{array}{c} J_{\mathrm{NADH},\mathrm{gly}}=2J_{G6P}\left(1-p_{L}\right)\; \end{array}$$

(4) $$\begin{array}{c} J_{\mathrm{pyr}}=2J_{G6P}\left(1-p_{L}\right)\; \end{array}$$

Finally, the ATP production rate due to oxidation of glycolysis-produced NADH molecules (*J*_ATP,NADH,gly_) is:

(5) $$\begin{array}{c} J_{\mathrm{ATP},\mathrm{NADH},\mathrm{gly}}=R_{P/O,\mathrm{gly}}\;J_{\mathrm{NADH},\mathrm{gly}} \end{array}$$

where *R*_P/O,gly_ is the P/O ratio for the glycerol-phosphate shuttle. The rest of the pyruvate ($1-p_{L}$) is transported into mitochondria, where it enters the TCA cycle. There, a fraction (*p*_TCA_) is used for the replenishment of TCA cycle intermediates that have been extracted for biosynthesis (anaplerotic reactions). Since anaplerosis was not detected in the experimental setting [31], we assume *p*_TCA_ = 1. The reactions of pyruvate dehydrogenase (PDH) and TCA cycle yield 4 molecules of NADH, 1 molecule of FADH_2,_ and 1 GTP molecule, the sum of which is energetically equal to the production of 5 molecules of NADH (for an extended explanation of this simplification, see [38,41]). The rate of NADH production due to the pyruvate oxidation (*J*_NADH,pyr_) is given by:

(6) $$\begin{array}{c} J_{\mathrm{NADH},\mathrm{pyr}}=5p_{\mathrm{TCA}}\left(1-p_{L}\right)J_{\mathrm{pyr}}\; \end{array}$$

The ATP production rate due to oxidation of pyruvate-derived NADH molecules (*J*_ATP,NADH,pyr_) is given by:

(7) $$\begin{array}{c} J_{\mathrm{ATP},\mathrm{NADH},\mathrm{pyr}}=R_{P/O,\mathrm{pyr}}\;J_{\mathrm{NADH},\mathrm{pyr}}\; \end{array}$$

where *R*_P/O,pyr_ is the P/O ratio for pyruvate oxidation. The rate of oxygen consumption at the electron transport chain (ETC) due to glucose oxidation ($J_{O_{2},G}$) is calculated by:

(8) $$\begin{array}{c} J_{O_{2},G}=\frac{1}{2}\left(J_{\mathrm{NADH},\mathrm{pyr}}+J_{\mathrm{NADH},\mathrm{gly}}-p_{L}J_{\mathrm{pyr}}\right)\; \end{array}$$

The alpha cell’s net oxygen consumption rate ($J_{O_{2}}$) is modeled by:

(9) $$\begin{array}{c} J_{O_{2}}=k_{O_{2}}J_{G6P}+J_{O_{2},0}\; \end{array}$$

where $J_{O_{2},0}$ is the base oxygen consumption and $k_{O_{2}}$ is the constant of proportionality. Values of parameters $k_{O_{2}}$ = −0.2 and $J_{O_{2},0}$ = 16 μM s^−1^ were determined by fitting the model-produced ATP concentration to the experimental data, which shows the elevation of ATP by 10% due to the increase in glucose concentration from 1 to 5 mM [8]. The negative value of $k_{O_{2}}$ is also consistent with greater oxygen consumption in hypoxic conditions, matching the elevation of FFA oxidation [38] since the P/O ratio for FFA oxidation is lower than for glucose oxidation [42,43].

In addition to the glucose oxidation pathway, the model incorporates the FFA oxidation pathway, which also contributes to ATP production. Similar to the pyruvate, FFAs enter mitochondria where they are broken down to acetyl-CoA molecules via beta-oxidation. Catabolism of glucose-derived pyruvate molecules and FFA-derived acetyl-CoA molecules in the TCA cycle yields NADH, FADH_2_, GTP, and CO_2_ molecules. Rather than the rate of FFA entry into the beta-oxidation reactions, the input to the model is the rate of oxygen consumption, for which the experimental data are more widely available. Assuming that the flux of oxygen entering the cell is fully consumed by the ETC for the oxidation of NADH and other reducing equivalents, which are by assumption produced solely by the glucose and FFA oxidation, the NADH production rate is calculated as the difference between the net rate of oxygen consumption ($J_{O_{2}}$) and oxygen consumption due to the glucose oxidation ($J_{O_{2},G}$). Therefore, the rate of NADH production due to the FFA oxidation (*J*_NADH,FFA_) is given by:

(10) $$\begin{array}{c} J_{\mathrm{NADH},\mathrm{FFA}}=2\left(J_{O_{2}}-J_{O_{2},G}\right)\; \end{array}$$

Consequently, the ATP production rate due to oxidation of FFA-produced NADH molecules (*J*_ATP,NADH,FFA_) is given by:

(11) $$\begin{array}{c} J_{\mathrm{ATP},\mathrm{NADH},\mathrm{FFA}}=R_{P/O,\mathrm{FFA}}\;J_{\mathrm{NADH},\mathrm{FFA}} \end{array}$$

where *R*_P/O,FFA_ is the P/O ratio for the FFA oxidation.

In the steady-state approximation, intracellular concentrations of adenine nucleotides ([ADP] and [ATP]) are defined by the glucose-dependent ATP production rate (*J*_ATP_) and the rate of ATP hydrolysis by ATP-ases (*J*_ATPase_). *J*_ATP_ is given by:

(12) $$\begin{array}{c} J_{\mathrm{ATP}}=J_{\mathrm{ATP},\mathrm{gly}}+J_{\mathrm{ATP},\mathrm{NADH},\mathrm{gly}}+J_{\mathrm{ATP},\mathrm{NADH},\mathrm{pyr}}+J_{\mathrm{ATP},\mathrm{NADH},\mathrm{FFA}} \end{array}$$

It is generally assumed that *J*_ATPase_ increases with the cell’s energy state [44], which is modeled by:

(13) $$\begin{array}{c} J_{\mathrm{ATPase}}=b_{\mathrm{ATPase}}\left[\mathrm{ATP}\right] \end{array}$$

The parameter value *b*_ATPase_ is unambiguously determined by the range of ATP concentrations (see Equation (14)), which was previously measured for pancreatic alpha cells [30]. In the steady-state approximation, when ATP production rate (*J*_ATP_) is equal to the ATP hydrolysis rate (*J*_ATPase_), Equations (12) and (13) yield:

(14) $$\begin{array}{c} \left[\mathrm{ATP}\right]=\frac{J_{\mathrm{ATP}}}{b_{\mathrm{ATPase}}}\; \end{array}$$

and the ATP-to-ADP ratio (RAT) is defined by:

(15) $$\begin{array}{c} RAT=\frac{\left[\mathrm{ATP}\right]}{\left[\mathrm{ADP}\right]}=\frac{\left[\mathrm{ATP}\right]}{A_{\mathrm{tot}}-\left[\mathrm{ATP}\right]}\; \end{array}$$

The conductance of the K_ATP_ channel is ATP- and ADP-dependent. *g*_KATP_ is commonly modeled according to the exponential function of either [ATP] [45] or RAT [44]. The function of K_ATP_ conductance is modeled so that it matches values at 1 mM glucose (0.27 nS) and 6 mM glucose (0.20 nS) as measured experimentally [46]:

(16) $$\begin{array}{c} g_{\mathrm{KATP}}=A_{\mathrm{KATP}}\;e^{-B_{\mathrm{KATP}}\cdot \mathrm{RAT}}\; \end{array}$$

where *A*_KATP_ and *B*_KATP_ are best-fit constants. The ATP concentration directly affects K_ATP_ channels’ conductance, which is the input parameter of existing electrophysiological models [36]. K_ATP_ channel conductance serves as the link between metabolism and electrophysiology of the cell. Electrophysiological model couples K_ATP_ channel conductance to the hormone granule exocytosis via modulation of calcium channel influx through VDCC. The parameter values used in the equations presented in this subsection are given in Table 1.

### 2.2. The cAMP Signaling Component

In addition to the sensing of the energy state of the cell, the alpha cell possesses several additional energy-state-independent mechanisms for modulation of glucagon secretion. Here, we model the mechanisms involved in the modulation of sAC activity and consequent cAMP production. cAMP is a ubiquitous second messenger, the activator of effector proteins involved in proliferation, differentiation, secretion, contractility etc. [49]. cAMP effector proteins involve protein kinase A (PKA), exchange factor directly activated by cyclic AMP (EPAC), cyclic AMP responsive ion channels (CICs), and the Popeye domain-containing (POPDC) proteins.

Depending on the metabolic rate, the cell produces a flux of lactic acid and CO_2_ molecules, which manifests in changes in CO_2_ and HCO_3_^−^ concentrations and pH. We assume the concentrations to be only dependent on the flux of carbon dioxide ($J_{{\mathrm{CO}}_{2}}$) and lactic acid production (*J*_lac_). The equilibrium between these metabolites is maintained according to the Henderson–Hasselbalch equation of the bicarbonate buffer. In the continuation, a detailed description of the corresponding mathematical model is given.

### 2.3. CO_2_ and H^+^ as Signaling Molecules/Ions

The ratio of carbon dioxide production to oxygen consumption is estimated by the respiratory quotient (RQ). The RQ for glucose oxidation is 1. On the other hand, the RQ for FFA is approximately 0.7, resulting in lower CO_2_ production compared to O_2_ consumption. The carbon dioxide production rate ($J_{{\mathrm{CO}}_{2}}$) is then calculated by:

(17) $$\begin{array}{c} J_{{\mathrm{CO}}_{2}}=J_{O_{2},G}+0.7\left(J_{O_{2}}-J_{O_{2},G}\right)=J_{O_{2},G}+0.35\;J_{\mathrm{NADH},\mathrm{FFA}} \end{array}$$

where *J*_NADH,FFA_ is the FFA-derived NADH production rate. We assume that the majority of the pH change is due to the production of lactic acid during anaerobic glycolysis (*J*_lac_), defined by:

(18) $$\begin{array}{c} J_{\mathrm{lac}}=2p_{L}J_{G6P}\; \end{array}$$

Lactic acid accumulates in the cell during its high production rate and mostly dissociates into the conjugate base (lac^−^) and hydrogen ion (H^+^) under physiological pH conditions.

### 2.4. Fluxes through the Plasma Membrane

The CO_2_ flux produces a gradient in CO_2_ concentration between the source of CO_2_ (mitochondria) and the sink of CO_2_ (blood capillary), which is the driving force of CO_2_ elimination from the cell [50]. We model the concentration values for the intracellular compartment bounded by the plasma membrane (PM), while we assume constant basal values in the extracellular space. Movement of CO_2_ through the PM ($J_{{\mathrm{CO}}_{2},\mathrm{PM}}$) is modeled according to the passive diffusion kinetics:

(19) $$\begin{array}{c} J_{{\mathrm{CO}}_{2},\mathrm{PM}}=k_{{\mathrm{CO}}_{2},\mathrm{PM}}\left({\left[{\mathrm{CO}}_{2}\right]}_{\mathrm{in}}-{\left[{\mathrm{CO}}_{2}\right]}_{\mathrm{bas}}\right) \end{array}$$

where *k*_CO2,PM_ is the corresponding diffusion constant, [CO_2_]_in_ is intracellular CO_2_ concentration and [CO_2_]_bas_ is extracellular (constant) CO_2_ concentration.

On the other hand, H^+^ is generated by the anaerobic metabolism and is removed from the cell cytoplasm by several transporters such as monocarboxylate transporter (MCT), Na^+^/H^+^ exchanger (NHE), H^+^ ATPase, etc. For simplicity, we assume only joint removal of H^+^ and lactate by the MCT and model it as a gradient-dependent function:

(20) $$\begin{array}{c} J_{H,\mathrm{PM}}=\;k_{H^{+},\mathrm{PM}}({\left[H^{+}\right]}_{\mathrm{in}}-({\left[H^{+}\right]}_{\mathrm{bas}}) \end{array}$$

where $k_{H^{+},\mathrm{PM}}$ is the corresponding constant, [H^+^]_in_ is intracellular H^+^ concentration and [H^+^]_bas_ is extracellular (constant) H^+^ concentration.

Lastly, transport of bicarbonate in mammals is mostly facilitated by Cl^−^/HCO_3_^−^ exchangers (AEs), Na^+^-coupled transporters (NBCs) [51,52], and they are also expressed in islet cells [53,54]. While transport dynamics of bicarbonate might be relatively complex, involving membrane potential (electrogenic transporters) and concentrations of co-transported ions, we again assume a simplified gradient-dependent removal of bicarbonate:

(21) $$\begin{array}{c} J_{{\mathrm{HCO}}_{3}^{-},\mathrm{PM}}=k_{{\mathrm{HCO}}_{3}^{-},\mathrm{PM}}\left({\left[{\mathrm{HCO}}_{3}^{-}\right]}_{\mathrm{bas}}-{\left[{\mathrm{HCO}}_{3}^{-}\right]}_{\mathrm{in}}\;\right) \end{array}$$

where $k_{{\mathrm{HCO}}_{3}^{-},\mathrm{PM}}$ is the corresponding constant, [HCO_3_^−^]_in_ is the intracellular bicarbonate concentration, and [HCO_3_^−^]_bas_ is the extracellular (constant) bicarbonate concentration.

### 2.5. The Henderson–Hasselbalch Equilibrium

The metabolic CO_2_ (which is relatively stable with increasing glucose) is in equilibrium with the carbonic acid. The hydration of CO_2_ is greatly accelerated by the intracellular carbonic anhydrases (CA), which are expressed in human alpha cells. In particular, the glucagon colocalization of the cytoplasmic form of the isoenzyme CA I has been found in alpha cells [22]. Carbonic acid is diprotic, and the dissociation of one proton forms the bicarbonate ion:

(22) $$\begin{array}{c} {\mathrm{CO}}_{2}{+H}_{2}O\overset{\mathrm{fast}\;\mathrm{equilibrium}\;\left(\mathrm{CA}\right)}{\Leftrightarrow }H_{2}{\mathrm{CO}}_{3}\overset{\mathrm{fast}\;\mathrm{equilibrium}}{\Leftrightarrow }{\mathrm{HCO}}_{3}^{-}{+H}^{+}\; \end{array}$$

The second dissociation (producing CO_3_^2−^ and another proton) is ignored as it is insignificant at physiological pH levels [55]. A common approach for calculating reaction equilibrium is to derive the apparent reaction, taking into account that H_2_CO_3_ is, in fact, strong acid, and common analytical procedures cannot distinguish H_2_CO_3_ from CO_2_ [55]:

(23) $$\begin{array}{c} {\mathrm{CO}}_{2}{+H}_{2}O\overset{\mathrm{fast}\;\mathrm{equilibrium}}{\Leftrightarrow }{\mathrm{HCO}}_{3}^{-}{+H}^{+} \end{array}$$

Here, we consider the analytical CO_2_ as a weak acid. The derived equilibrium constant for the apparent reaction (Equation (23)) yields Equation (24) for intracellular concentrations of bicarbonate buffer metabolites:

(24) $$\begin{array}{c} K_{a}=\frac{{\left[{\mathrm{HCO}}_{3}^{-}\right]}_{\mathrm{in}}{\left[H^{+}\right]}_{\mathrm{in}}}{{\left[{\mathrm{CO}}_{2}\right]}_{\mathrm{in}}} \end{array}$$

Finally, the changes in intracellular concentration due to influx (production) and efflux rates are described by the system of equations:

(25) $$\begin{array}{c} \left[\begin{array}{c} \frac{d{\left[{\mathrm{HCO}}_{3}^{-}\right]}_{\mathrm{in}}}{\mathrm{dt}}\\ \frac{d{\left[H^{+}\right]}_{\mathrm{in}}}{\mathrm{dt}}\\ \frac{d{\left[{\mathrm{CO}}_{2}\right]}_{\mathrm{in}}}{\mathrm{dt}} \end{array}\right]=\left[\begin{array}{c} J_{{\mathrm{HCO}}_{3}^{-},\mathrm{PM}}-J_{\mathrm{IC}}\\ J_{\mathrm{lac}}-J_{H^{+},\mathrm{PM}}-J_{\mathrm{IC}}\\ J_{{\mathrm{CO}}_{2}}-J_{{\mathrm{CO}}_{2},\mathrm{PM}}+J_{\mathrm{IC}} \end{array}\right] \end{array}$$

where *J*_IC_ is the flux due to the interconversion of [HCO_3_^−^]_in_, [H^+^]_in_, and [CO_2_]_in_, according to Equations (22)–(24), and other fluxes are defined by Equations (17)–(21). In the steady-state approximation, Equations (24)–(25) yield a system of four equations with four variables, which can be solved numerically. We assume basal conditions to be the same as in the venous blood (values of [HCO_3_^−^]_bas_, [H^+^]_bas_, and [CO_2_]_bas_ are defined in Table 2).

### 2.6. Modeling cAMP Levels

The major sources of cAMP in mammalian cells are tmAC (AC1–9), which produce cAMP from ATP in response to adrenaline and incretins, and sAC (AC10), which are stimulated by Ca^2+^, HCO_3_^−^, and ATP. In human islets, mRNAs of AC1, 3, 5, 6, 8, 9, and 10 have been detected, but we lack alpha-cell-specific data [14]. The activity of human sAC obeys Michaelis–Menten kinetics [56]. The half-maximal effect (EC50) for sAC stimulation by NaHCO_3_ is ~11 mM, and the *K*_m_ for ATP is ~0.8 mM [17]. Experimental data suggest that the vast majority of cAMP is produced due to enhanced metabolism, and only a small fraction (<10%) is produced due to the calcium influx [23]. Since the concentration of ATP is relatively constant, the increase in cAMP is primarily contributed to the HCO_3_^−^, which is formed due to accelerated metabolism and CO_2_ output. It is important to note that sAC is directly stimulated by physiological levels of the bicarbonate anion [17]. Assuming that the changes of ATP are insignificant in alpha cells, the rate of cAMP production (*J*_sAC_) is described by:

(26) $$\begin{array}{c} J_{\mathrm{sAC}}=k_{\mathrm{sAC}}\frac{{\left[{\mathrm{HCO}}_{3}^{-}\right]}_{\mathrm{in}}}{{\left[{\mathrm{HCO}}_{3}^{-}\right]}_{\mathrm{in}}+K_{m,\mathrm{sAC}}}\; \end{array}$$

where *k*_sAC_ is the maximal rate of cAMP production and *K*_m,sAC_ is the Michaelis–Menten constant. Apart from the cAMP synthesis rate, cAMP concentration is also determined by its degradation. The cAMP is converted to AMP and phosphate by the enzyme phosphodiesterase (PDE). Alpha cells predominantly express PDE3 and PDE4. While the PDE4-selective inhibitor rolipram stimulates glucagon secretion at all glucose levels, the PDE3B-specific inhibitor cilostamide evokes a glucose dose-dependent increase in glucagon secretion, which is a result of insulin inhibition of PDE3B, driving degradation of cAMP and inhibition of PKA signaling [39]. PDE3 hydrolyzes both cGMP and cAMP with *K*_m_ values between 0.1–0.8 μM, but the *V*_max_ for cAMP hydrolysis is 4–10 times higher than *V*_max_ for cGMP hydrolysis [57]. Rolipram-inhibited PDE4, however, has a high affinity for cAMP and a *K*_m_ value of 4.4 μM [58]. These values roughly match the model by [59], in which *K*_m_ for PDE is set to 3 μM. Therefore, we model the PDE activity (*J*_PDE3B_ and *J*_PDE4_ for PDE3B and PDE4, respectively) by:

(27) $$\begin{array}{c} J_{\mathrm{PDE}3B}=k_{\mathrm{PDE}3B}\frac{\left[\mathrm{cAMP}\right]}{\left[\mathrm{cAMP}\right]+K_{m,\mathrm{PDE}3B}} \end{array}$$

(28) $$\begin{array}{c} J_{\mathrm{PDE}4}=k_{\mathrm{PDE}4}\frac{\left[\mathrm{cAMP}\right]}{\left[\mathrm{cAMP}\right]+K_{m,\mathrm{PDE}4}} \end{array}$$

where *k*_PDE3B_ and *k*_PDE4_ are the maximal rates of cAMP hydrolysis, and *K*_m,PDE3B_ and *K*_m,PDE4_ are the Michaelis–Menten constants for PDE3B and PDE4, respectively. [cAMP] is the cAMP concentration, which is calculated by solving the equation:

(29) $$\begin{array}{c} r_{\mathrm{sAC}}J_{\mathrm{sAC}}=r_{\mathrm{PDE}3B}J_{\mathrm{PDE}3B}+r_{\mathrm{PDE}4}J_{\mathrm{PDE}4}\; \end{array}$$

where *r*_sAC_ is the proportion of maximal sAC activity relative to the maximal PDE activity, and *r*_PDE3B_ and *r*_PDE4_ is the maximal PDE3B and PDE4 activity relative to the maximal PDE activity, respectively. Both *r*_PDE3B_ and *r*_PDE4_ are set to 0.5 (assuming 50% contribution of cAMP hydrolysis by PDE3B and PDE4). The factor *r*_sAC_ is set to 1.2, fitting the representative cAMP values.

### 2.7. Direct and Indirect Action of cAMP on Exocytosis

The cAMP signaling pathway acts via two distinct mechanisms; PKA-dependent and PKA-independent (EPAC) pathways. Both have an amplifying effect on glucagon secretion by several mechanisms. cAMP has been reported to influence calcium signaling, mainly by mobilizing Ca^2+^ current from intracellular stores and by PKA-dependent activation of L-type VDCC in alpha cells [10]. The action of cAMP on various intracellular mechanisms is modeled by

(30) $$\begin{array}{c} f_{\mathrm{cAMP}}=\frac{\left[\mathrm{cAMP}\right]-{\left[\mathrm{cAMP}\right]}_{\min}}{{\left[\mathrm{cAMP}\right]}_{\max}-{\left[\mathrm{cAMP}\right]}_{\min}} \end{array}$$

where [cAMP]_min_ and [cAMP]_max_ are the minimal and maximal values of cAMP, respectively. In alpha cell, increasing glucose from 3 to 20 mM reduces cAMP concentration by approximately 35% [10,23,39].

It has been shown that glucose regulates cAMP by a direct effect on alpha cells and that the signaling effect is more important than changes in calcium [10]. Based on the study by Yu et al. [23], we assume the proportion of the K_ATP_ channels and the direct effect of Ca^2+^ on the exocytosis to be ~40% and the contribution of cAMP ~60%. Furthermore, we assume that the increase in calcium has a negligible effect on ATP. This is in agreement with previous studies, revealing that the cAMP signaling is independent of intracellular calcium [39] or that the effect of calcium is minimal [23]. The parameter values used in the equations presented in this subsection are given in Table 2.

### 2.8. The Glucagon Secretion Component

For the glucagon secretion, we used the model introduced by Montefusco and Pedersen [36]. The model’s input parameter is *g*_KATP_, which is yielded by the metabolic component of the computational model. The electrophysiological model produces glucose-dependent membrane potential oscillations. An increase in glucose concentration reduces voltage amplitude and the corresponding Ca^2+^ influx through P/Q-type VDCC, reducing glucagon exocytosis (for more details, see Supplementary Materials, Figure S1). We modified the model by taking into account the parameter *f*_cAMP_, which modifies glucagon secretion directly and indirectly via modulation of calcium currents. The additional features, which are added to the initial model, present a more in-depth portrayal of alpha cell’s metabolic and signaling behavior in response to increased glucose concentration. More precisely, we introduce the direct effect of cAMP on glucagon secretion. The total amount of glucagon secretion is comprised of three contributions: the cumulative P/Q- and L-type microdomain-dependent exocytosis (*GS*_P/Q_ and *GS*_L_, respectively), and the cumulative secretion depending on the sub-membrane Ca^2+^ concentration (*GS*_m_) (for details, see [36]). We assume the cAMP mainly affects the granules in the L- and P/Q-channel microdomains by introducing parameter *f*_direct_:

(31) $$\begin{array}{c} GS=f_{\mathrm{direct}}\left(GS_{P/Q}+GS_{L}\right)+GS_{m}\; \end{array}$$

where

(32) $$\begin{array}{c} f_{\mathrm{direct}}=\left(1-k_{\mathrm{direct}}\left(1-f_{\mathrm{cAMP}}\right)\right)\; \end{array}$$

and where *k*_direct_ represents a relative effect of cAMP on exocytosis due to P/Q- and L-type calcium channel influx.

Secondly, cAMP acts indirectly by modulating Ca^2+^ currents via VDCC. We model this indirect effect by introducing parameter *f*_indirect_, which we incorporate into the equation for the membrane potential dynamics:

(33) $$\begin{array}{c} \frac{dV}{dt}=\frac{-\left(f_{\mathrm{indirect}}\left(I_{\mathrm{CaL}}+I_{\mathrm{CaP}/Q}+I_{\mathrm{CaT}}\right)+I_{\mathrm{Na}}+I_{K}+I_{\mathrm{KATP}}+I_{\mathrm{KA}}+I_{L}+I_{\mathrm{SOC}}\right)}{C_{m}}\; \end{array}$$

where

(34) $$\begin{array}{c} f_{\mathrm{indirect}}=\left(1-k_{\mathrm{indirect}}\left(1-f_{\mathrm{cAMP}}\right)\right)\; \end{array}$$

and where the *k*_indirect_ represents the relative effect of cAMP on the Ca^2+^ increase. Both parameters *k*_direct_ and *k*_indirect_ influence glucagon secretion due to their coupling to the *f*_cAMP_. To assess relative contributions of both parameters to the glucagon secretion, stability analysis was performed. The model was stable under perturbations of parameters (see Supplementary Materials, Figure S2).

Lastly, the input of the metabolic model into the secretion model is *g*_KATP_, which determines the flux through the K_ATP_ channels in Equation (16) by:

(35) $$\begin{array}{c} I_{\mathrm{KATP}}=g_{\mathrm{KATP}}\left(V-V_{K}\right)\; \end{array}$$

where *V*_K_ is the Nernst potential for potassium ions. All other model parameters in the alpha cell glucagon secretion model take on the same value as in the original manuscript by [36]. The glucose-dependency of the leak current (*g*_L_) is modeled by:

(36) $$\begin{array}{c} g_{L}=g_{L,\min}+\frac{g_{L,1/2}{}^{n_{h}}}{g_{L,1/2}^{n_{h}}+g_{\mathrm{KATP}}^{n_{h}}}\Delta g_{L}\; \end{array}$$

The values of the model parameters used in Equations (31)–(36) are given in Table 3.

## 3. Results

**Figure 2:**
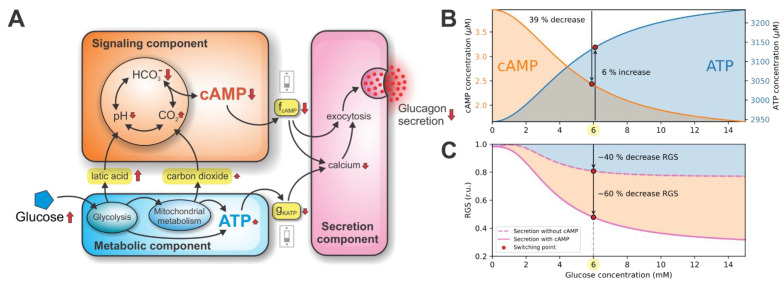
Metabolic and signaling components providing a double switch of glucagon secretion. (**A**) A schematic representation of the model dynamics. Interconnected metabolic and signaling components both contribute to the secretion component via *g*_KATP_ and *f*_cAMP_ coupling parameters, respectively. (**B**) Glucose-dependent cAMP and ATP concentrations. The cAMP-driven signaling switch is initiated by the 39% decrease in cAMP concentration. On the other hand, the energy-driven switch of glucagon secretion is initiated by the modest 6% increase in ATP concentration. (**C**) The glucose-dependent RGS. At the switching point, metabolic and signaling components contribute 40% and 60% to the RGS, respectively.

The computational model, presented in Paragraph 2, enables simulation of glucagon secretion from pancreatic alpha cells upon glucose stimulation. The metabolic pathways, which consist of glucose oxidation, FFA oxidation, and the electron transport chain (ETC), are responsible for the production of ATP (Equations (1)–(16) of the model). In parallel, the cAMP signaling pathway (Equations (17)–(30) of the model) results in the production of cAMP. Both pathways activate the secretory mechanisms for glucagon secretion (Equations (31)–(36) of the model). Qualitatively, the model dynamics are schematically presented in Figure 2A. The model predicts that in the metabolic component (depicted in blue), the increased glucose metabolism leads to changes in ATP concentration. Complementary to the rise in ATP concentration, the metabolic component yields an increased rate of lactic acid (produced by glycolysis) and carbon dioxide (produced by the mitochondrial metabolism). Both metabolites are the inputs to the signaling component of the model (depicted in orange), influencing the processes leading to the modulation of cAMP concentration. Upon glucose stimulation, both metabolic and signaling components act on the secretion component (depicted in magenta). The increased ATP concentration acts on the secretion component via parameter *g*_KATP_, decreasing the Ca^2+^ concentration and consequently switching the glucagon secretion off. Similarly, the decreased cAMP concentration acts on the secretion component via parameter *f*_cAMP_, again decreasing Ca^2+^ concentration and additionally directly inhibiting glucagon exocytosis.

The qualitative results in Figure 2A show that the glucagon secretion is turned on/off by the two distinct switching mechanisms. The ATP-driven metabolic switch is activated by the rise in the net ATP concentration, while the cAMP-driven signaling switch is activated by the drop in cAMP concentration. The synergistic action of both switches acts as a double switch of glucagon secretion. The quantitative model predictions of the double switch acting on glucagon secretion are presented in the following sections.

### 3.1. Double Switch for Glucose-Induced Glucagon Secretion

Intracellular ATP and cAMP concentrations are the decisive molecules in modulating the double switch for glucose-induced glucagon secretion. Their concentrations related to the glucose stimulation are shown in Figure 2B. At the glucose concentration of 6 mM (referred to as the switching point), an increase in plasma glucose causes a relatively modest, 6% increase in ATP concentration. This increase in ATP concentration results from the intensified glucose metabolism. The rise in ATP concentration acts on the metabolic switch by slightly lowering the conductance of K_ATP_ channels (*g*_KATP_), indirectly diminishing glucagon exocytosis by reducing calcium influx. Simultaneously, an increase in plasma glucose at the switching point also causes a significant ~39% drop in cAMP concentration, resulting from the increase in lactic acid and carbon dioxide production. High cAMP levels at glucose concentrations below the switching point act on the signaling switch via model parameter *f*_cAMP_, which represents the relative cAMP concentration (occupying values between 0 and 1, see Equation (30)). The cAMP affects glucagon secretion directly (by promoting exocytotic mechanisms) and indirectly (by maintaining high intracellular calcium concentration). Above the switching point, the cAMP concentration is markedly reduced, resulting in diminished glucagon secretion due to the signaling component.

The computational evaluation of contributions to glucagon secretion due to metabolic and signaling components are shown in Figure 2C. During hypoglycemia, the relative glucagon secretion (RGS) is maintained at high levels. Raising glucose to the switching point decreases the RGS to approximately 40% of the maximal value. Increasing glucose above the switching point has a modest effect on the RGS. To estimate the contributions of both components, we set the parameter *f*_cAMP_ to 0, which decoupled the signaling and secretion components (see Figure 2A). Therefore, only the effect of the ATP-driven metabolic switch on the RGS is observed.

In the next sections, we provide a detailed description of how the ATP-driven metabolic switch and the cAMP-driven signaling switch operate.

### 3.2. ATP-Driven Metabolic Switch

The metabolic part of the double switch is ATP-driven; above the switching point, ATP concentration reaches the critical value, which switches off the glucagon secretion. ATP is generated by the glucose and FFA oxidation pathways. The rates of both pathways are glucose-dependent, which modulates ATP production. In this section, we present the ATP-generating mechanisms, which are responsible for the glucose-dependent rise in ATP concentration, and quantitatively assess their contributions.

**Figure 3:**
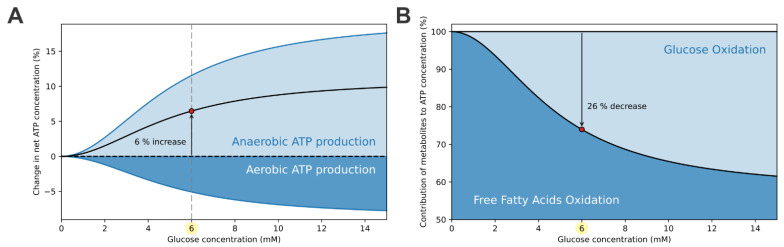
Means of ATP production in alpha cells. (**A**) The net ATP concentration (black curve) increases by approximately 6% at the switching points. This increase is mainly due to the anaerobic (glycolytic) ATP production (light blue curve), while the aerobic (mitochondrial) ATP production (dark blue curve) opposes the increase. (**B**) In hypoglycemic conditions, the majority of ATP is produced by the oxidation of FFAs. With rasing glucose levels and consequent increase in glycolytic flux, a fraction of FFA oxidation is replaced with glucose oxidation. At the switching point, FFA oxidation is decreased by approximately 26%.

The computational model separates the anaerobically- and aerobically-produced ATP. ATP is produced anaerobically during glycolysis, the rate of which is glucose dependent (Equation (1)). On the other hand, aerobic ATP generation occurs at the ETC as a result of glucose and FFA oxidation pathways. The activity of the ETC and consequent aerobic ATP production is proportional to the rate of oxygen consumption (Equation (9)). The contributions of aerobic and anaerobic processes to the ATP concentration are shown in Figure 3A. The net ATP concentration relative to the ATP concentration at the starting point (0 mM glucose concentration) increases by approximately 6% at the switching point. This change results from two opposing effects. The isolated glucose-dependency of relative ATP concentration due to the anaerobic rate of ATP production (light blue curve) shows a ~11% increase at the switching point. On the other hand, the isolated glucose-dependency of ATP concentration due to the aerobic rate of ATP production (dark blue curve) shows a ~5% decrease. While the anaerobic and aerobic metabolisms have conflicting effects on the ATP concentration, the anaerobic (glycolytic) metabolism prevails, resulting in a modest increase in net ATP concentration.

The elevation of ATP concentration is modest. Preservation of ATP production at high levels is accomplished by maintaining high rates of FFA oxidation. Figure 3B presents the relative contributions of FFA oxidation and glucose oxidation to ATP production. During hypoglycemia, the vast majority of ATP is produced by FFA oxidation. With the elevation of glycolytic flux, using up some of the mitochondrial capacity, the aerobic metabolism shifts toward glucose oxidation, which is at supraphysiological conditions responsible for up to 40% of ATP production.

These results demonstrate that the metabolic switch, which switches off the glucagon secretion at higher glucose concentrations, dependents mostly on the anaerobic (glycolytic) glucose metabolism. The modest increase in the net ATP concentration at the switching point, shown in Figure 3, is sufficient to switch the glucagon secretion off. Under hypoglycemia, high electrical activity maintains high calcium concentration and preserves high RGS (see Figure 2A). However, with raising glucose levels, decreased *g*_KATP_ reduces the calcium concentration, which corresponds with lower RGS.

### 3.3. cAMP-Driven Signaling Switch

**Figure 4:**
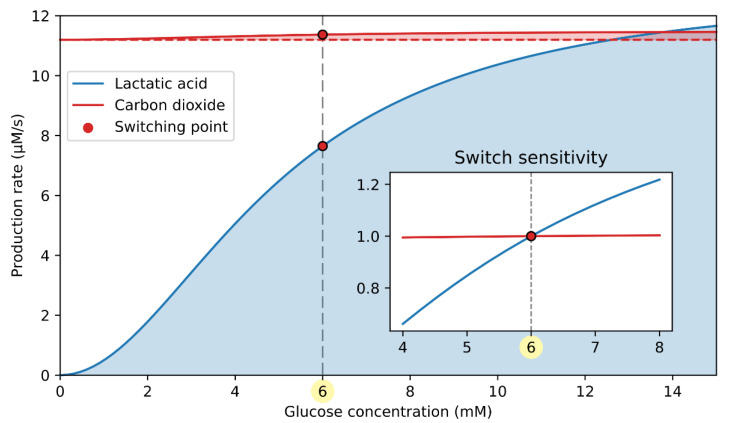
Lactic acid and carbon dioxide production rate in dependence on glucose concentration. CO_2_ production rate only slightly increases with glucose, making it much less important for the cAMP-driven signaling switch. The inset shows the switch sensitivity, indicating a relative influence of the catabolites on the cAMP-driven switch. The switch sensitivity of lactic acid and carbon dioxide production is calculated as the absolute value divided by the lactic acid and carbon dioxide production at the switching point (6 mM glucose), respectively.

As shown in Figure 2A, the signaling part of the double switch is cAMP-driven. The computational model predicts that the cAMP concentration drops by 39% at the switching point (Figure 2B), which efficiently contributes to the lower glucagon secretion (Figure 2C). The switching-off mechanism is initiated by changes in intracellular lactic acid and carbon dioxide production rates, induced by the rise in glucose concentration, as schematically presented in Figure 2A. The production rate of lactic acid and carbon dioxide in relation to the glucose concentration is quantified in Figure 4. Both lactic acid and CO_2_ production rates increase with the rise in stimulatory glucose concentrations.

During hypoglycemia, the primary metabolic source for ATP production is the FFAs. Since FFAs are highly reduced molecules, their respiratory quotient (RQ), defined as the ratio between produced CO_2_ and consumed O_2,_ is low (RQ≐0.7). Therefore, the yield of CO_2_ due to FFA oxidation is ~30% lower compared to the rate of oxygen consumption. With rising glucose concentration, the glucose-to-FFA oxidation ratio increases, and glucose (RQ=1) becomes the primary metabolic fuel during hyperglycemia. The CO_2_ output due to glucose oxidation is similar to the rate of oxygen consumption. As a result, the CO_2_ production rate is accelerated despite the decrease in oxygen consumption (see Equation (9)). Compared to CO_2_, however, the change in lactic acid concentration is far more pronounced. The lactic acid output is proportional to the glycolytic flux (see Equation (18)), which is greatly accelerated with the rise in glucose concentration. In the inset of Figure 4, the effective contribution of the lactate and CO_2_ to switching glucagon secretion off is characterized by the switch sensitivity, representing the substrate production rate relative to that at the switching point. It is evident that the switch sensitivity is significantly higher for lactic acid than for carbon dioxide, implicating a greater role of lactic acid in modulating the cAMP-driven signaling switch.

**Figure 5:**
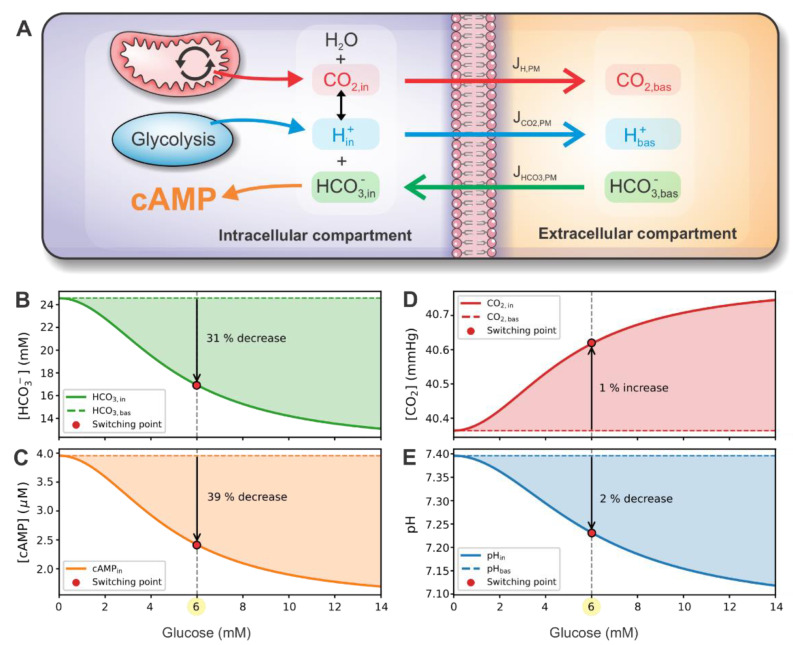
Metabolite concentration and fluxes in intracellular and extracellular compartments. (**A**) Schematic representation of compartment dynamics. CO_2_ and H^+^ are produced by mitochondria and glycolysis, respectively. Both metabolites are in equilibrium with HCO_3_^−^ according to the Henderson–Hasselbalch equation. HCO_3_^−^ concentration influences the production of cAMP. CO_2_, H^+^, and HCO_3_^−^ can move through the plasma membrane due to gradients between intracellular and extracellular compartments. In the extracellular compartment, metabolite values are constant. Panels (**B**–**E**) present glucose-dependent intracellular HCO_3_^−^, cAMP, CO_2_, and pH concentrations, respectively. Panels also show the relative change of concentration at the switching point compared to the basal value.

The glycolysis-produced lactic acid and the mitochondrial CO_2_ enter the cytosol, where they induce a shift in the bicarbonate buffer equilibrium, as schematically presented in Figure 5A. The bicarbonate buffer system involves the balance of H^+^ (pH), CO_2_, and HCO_3_^−^. In the presence of CA, the system is kept in rapid equilibrium, which is maintained by the interconversion according to the Henderson–Hasselbalch equation (see Equations (22)–(23)). The model prediction for HCO_3_^−^ concentration is presented in Figure 5B. In the extracellular compartment, bicarbonate concentration is kept constant, while intracellularly, it decreases by approximately 31% at the switching point. The decreased intracellular bicarbonate concentration has an inhibiting effect on cAMP production rate via reduced stimulation of sACs. As shown in Figure 5C, the intracellular cAMP concentration drops by approximately 39% at the switching point.

To further illuminate the operation of the signaling switch, we present the intracellular CO_2_ concentration and pH, which, via Henderson–Hasselbalch equation, modulate the imperative HCO_3_^−^ concentration. With the H^+^ production rate being highly glucose-dependent compared to the CO_2_ production rate (see Figure 4), a significant proportion of HCO_3_^−^ and H^+^ is converted to CO_2_, resulting in lower HCO_3_^−^ concentration. This way, the over-acidification of intracellular fluid is also prevented. Intracellular CO_2_ concentration and pH are presented in Figure 5D,E. At the switching point, CO_2_ concentration only slightly (~1%) increases, and pH drops by approximately 2%.

The plasma membrane, which separates the intracellular and extracellular compartments, allows for the transition of all three metabolites. Metabolites are excreted from the cell following the diffusion kinetics (see Equations (19)–(21)). In the extracellular compartment, metabolite values are kept constant, having the same values as in the venous blood. By maintaining all metabolite concentrations constant, the extracellular compartment acts as a sink to CO_2_ and H^+^ fluxes and the source of HCO_3_^−^.

The cAMP regulation dynamics appear to be crucially dependent on the metabolite concentrations involved in the bicarbonate buffer, particularly on its pH. The acidification of intracellular fluid during high metabolic activity shifts the Henderson–Hasselbalch equilibrium toward the higher CO_2_ production, resulting in lower intracellular HCO_3_^−^ concentration. To maintain the steady state, the influx of bicarbonate is accelerated following the drop in intracellular HCO_3_^−^ concentration. Since the CO_2_ production rate is not significantly glucose-dependent, the cAMP-driven signaling switch essentially senses the pH levels of the intracellular space, induced by the increased glycolytic activity. Being independent of the membrane potential, which is fundamental for the ATP-sensing metabolic switch, the cAMP-dependent signaling switch ensures additional reliability in controlling glucagon secretion.

## 4. Discussion

Glucagon, secreted from pancreatic alpha cells, is together with insulin, a crucial hormone regulating glucose homeostasis. Due to the importance of glucagon to prevent life-threatening hypoglycemia, several mechanisms are likely to be involved in the sensing of plasma glucose concentration. The here introduced double switch for glucagon secretion illustrates a synergistic action of two important mechanisms. The first part, the ATP-driven metabolic switch, depends on the catabolism of glucose and FFAs, being particularly relevant during hypoglycemic conditions to provide basal ATP levels. With the increased levels of glucose, glycolysis provides the required elevation in ATP concentration resulting in a slight reduction of K_ATP_ channel conductance and the consequent lower glucagon exocytosis. The second mechanism, the cAMP-driven signaling switch, is closely related to the metabolic processes and particularly depends on the lactate production in the process of glycolysis. The lower pH during high glycolytic activity results in a reduction of cAMP levels and glucagon exocytosis. Both parts of the here introduced double switch act synergistically, providing more reliable control of modulating glucagon secretion, and both relaying on the anaerobic nature of alpha cells via glycolytic ATP and lactate production. The mainly anaerobic fate of glucose in alpha cells was supported by several experimental findings [31,63,64], but it has been unclear how the preference for anaerobic glycolysis is connected to the glucose-dependent glucagon secretion.

Apart from glucose and FFAs, other nutrients are involved in alpha cell’s metabolic and signaling pathways. In particular, amino acids such as alanine and arginine are important stimuli for glucagon secretion [65]. An increase of amino acid concentration, acting on alpha cells, is followed by the increased glucagon output [66,67]. This phenomenon is observed in both in vitro and in vivo experiments. Moreover, the relationship between the plasma amino acid concentration and glucagon concentration is reciprocal—glucagon also modulates amino acid metabolism at the systemic level [7,65]. There is evidence of the liver-alpha-cell axis, where glucagon is a crucial regulator of amino acid homeostasis and loss of glucagon signaling impairs hepatic amino acid catabolism [68]. However, the role of amino acids in energy production seems to be less relevant since alpha cells preferentially use glucose and FFAs, not amino acids, for ATP production during hypoglycemia. In a similar manner, the energy production to sustain gluconeogenesis in hepatocytes during low glucose levels is fueled by FFA oxidation rather than amino acid catabolism [69]. The implementation of the amino acid contribution to the bioenergetics of the alpha cells is therefore much less reasonable since their involvement would add another layer of complexity to the alpha cell’s metabolism.

Considering that the fate of glucose in alpha cells is mostly directed toward lactic acid production, the intracellular space must undergo some degree of acidification. To some extent, the decrease in pH levels during hyperglycemia was observed in recent experiments [23,70], but the acidification is probably less pronounced due to compensation by the bicarbonate buffer system and excretion of protons from the intracellular space. It was also proposed that intracellular acidification can emerge from inhibition of the Na^+^/H^+^ antiport, which may result from Na^+^ accumulation due to Na^+^-coupled glucose entry [70]. Stimulatory pH was associated with glucagon secretion by several studies [71,72]. In one in vivo study on rats, the alkalosis of extracellular fluid surrounding the islet cells produced a transient although short-lasting increase in glucagon output during the presence of 2.3 mM glucose [71]. Specifically, the change in pH from 7.4 to 7.8 resulted in a brief three-fold increase in glucagon secretion. However, despite relatively low observed intracellular acidification, the decrease in intracellular HCO_3_^−^ concentration due to Henderson–Hasselbalch equilibrium may be substantial [73], consequently reducing cAMP levels due to a decrease in sAC stimulation.

For endocrine pancreatic cells, it is still not completely clear to what extent the elevated cAMP levels result from the increase in production by sAC or tmAC. In pancreatic beta cells, the distinct effects were studied by specific pharmacological inhibition of sAC and tmAC. It was found that tmACs are mainly responsible for basal cAMP production while sAC is mainly involved in the glucose-induced component [15]. The results could, however, be misleading due to the deleterious effects of the sAC inhibitor KH7 on beta-cell metabolism [74]. For alpha cells, the implication of the sAC to glucagon secretion remains to be tested experimentally. In contrast to beta cells, the fraction of sAC contribution to the total cAMP levels when considering both intrinsic and paracrine effects is probably much lower in alpha cells, considering that Ca^2+^ and ATP negligibly contribute to a glucose-dependent decrease in sAC stimulation. Additionally, since the cAMP concentration is most commonly measured using ratiometric cAMP sensors [10,23,39,74,75], absolute cAMP values presented in the model are uncertain. However, the cAMP concentration range in the model can be easily scaled without significant effect on the downstream model equations. Partly indicative of the absolute cAMP levels is the PDE kinetics, since the cAMP values should be far below the enzyme’s saturation point to ensure the ability of PDEs to control cAMP values when paracrine signals via tmACs further increase cAMP concentration. Despite the uncertainty of the absolute cAMP levels, our model results are in agreement with the relative cAMP concentration measurements, which suggest a glucose-dependent decrease by approximately 50% [10,23,39,75].

The separate consideration of the glucagon secretion double switch consisting of a metabolic and a signaling part provides better insight into the intrinsic mechanisms of alpha cell’s intracellular processes. The metabolic part contributing ~40% to glucagon secretion is tightly linked with calcium, which is consistent with the observations that calcium is less important in alpha cells than for beta cells, where it is the primary signal for insulin exocytosis [14]. On the other hand, evidence on the cAMP’s strong implication in glucagon secretion [10,14,23] is consistent with the cAMP signaling part of the double switch, contributing ~ 60% of the net effect. In the present model, however, several simplifications regarding the cAMP pathways have been made, leaving possibilities for future more precise modeling of the downstream cAMP targets, in particular PKA and Epac. Additionally, modeling of alpha cells in the islet setting requires the inclusion of glucose-dependent action of paracrine signals and juxtracrine factors, in particular from neighboring beta and delta cells [4,5,40,76,77,78], which represents a promising approach of investigating their contribution to cellular function as a whole, but introduces a new level of complexity.

## Figures and Tables

**Table 1:** Parameter Values for the Metabolic Part of the Computational Model.

Parameter	Value	References
*J*_max_	7.2 μM s^−1^	[38,47], fitted according to [31]
*K*_m_	5 mM	
*p*_TCA_	1	[38,47], estimated according to [31]
*p*_L_	0.9	[38,47], estimated according to [31,41]
*R*_P/O,G_	2.5	[48]
*R*_P/O,FFA_	2.3	
$k_{O_{2}}$	−0.2	[38,47], fitted to match relative ATP elevation [8]
$J_{O_{2},0}$	16 μM s^−1^	
*b*_ATPase_	0.025 s^−1^	Estimated to match ATP range [30]
*A*_tot_	4000 μM	[30,38]
*A*_KATP_	0.65 nS	K_ATP_ conductance constants are obtained by fitting values at 1 mM glucose (0.27 nS) and 6 mM glucose (0.20 nS) as measured experimentally [46], cf. [44]
*B*_KATP_	0.21	

**Table 2:** Parameter Values for the Signaling Part of the Model.

Parameter	Value	References
*K*_a_	10^−6.1^ M	Known dissociation constant [60]
[CO_2_]_bas_	40 mmHg = 1.232 mM	Typical values for venous blood [60,61]
[H^+^]_bas_	10^−7.4^ M	
${\left[{\mathrm{HCO}}_{3}^{-}\right]}_{\mathrm{bas}}=\frac{K_{a}{\left[{\mathrm{CO}}_{2}\right]}_{\mathrm{bas}}}{{\left[H^{+}\right]}_{\mathrm{bas}}}$	24.6 mM	
$k_{{\mathrm{HCO}}_{3}^{-},\mathrm{PM}}$	1 s^−1^	Fitted to match the minimal drop in pH [23] and physiological intracellular ranges in CO_2_ and HCO_3_^−^ concentrations [60]
$k_{H^{+},\mathrm{PM}}$	10^6^ s^−1^	
$k_{{\mathrm{CO}}_{2},\mathrm{PM}}$	10^3^ s^−1^	
*K*_m,sAC_	11 mM	[17]
*K*_m,PDE3B_	0.4 µM	[57]
*K*_m,PDE4_	4.4 µM	[58]
*r*_sAC_	1.0	Fitted to match the representative absolute cAMP values in islet cells [59]
*r*_PDE3B_	0.5	Assumed 50% of overall PDE activity [39,62]
*r*_PDE4_	0.5	

**Table 3:** Parameter Values for the Glucagon Secretion Part of the Model.

Parameter	Value	References
*k*_indirect_	0.05	Sensitivity analysis (see Supplementary Materials, Figure S2)
*k*_direct_	0.6	
*g*_L,min_	0.20 nS	[36]
*g*_L,1/2_	0.25 nS	
*n*_h_	9	[38]
Δ*g*_L_	0.04 nS	

## Data Availability

The source code for the computational model is available as a GitHub repository (https://github.com/janzmazek/Double-Switch-Model, accessed on 10 April 2021).

## Supplementary Materials

The following are available online at https://www.mdpi.com/article/10.3390/cells10040896/s1, Supplementary Materials, Figure S1. Characteristic dynamics of the secretion component, Supplementary Materials, Figure S2. Sensitivity analysis of parameters *k*_direct_ and *k*_indirect_.
