# Supplementary material for: Role of cAMP in Double Switch of Glucagon Secretion
Source: Cells. 2021 Apr 14;10(4):896. doi: 10.3390/cells10040896 (PMC8070687; doi:10.3390/cells10040896)
Supplement: Supplementary file 1 [file cells-10-00896-s001.pdf]

Supplement

Calcium dynamics

The secretion component's temporal dynamics is predominantly governed by the model introduced by [1]. Characteristic time courses of calcium currents, relative glucagon secretion (RGS) rates, and membrane voltage for two stimulatory glucose concentrations are shown in Fig S1.

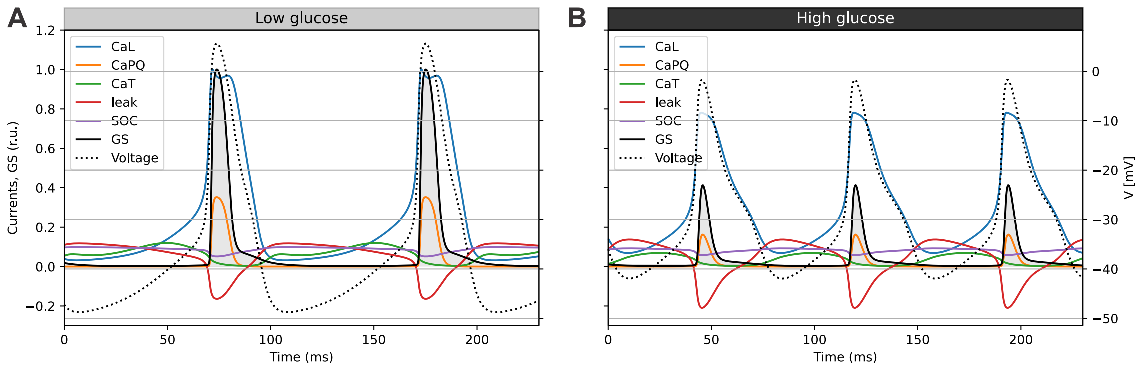

Fig S1: Characteristic dynamics of the secretion component. Graphs show normalized calcium currents (I_{CaL}, I_{CaPQ}, I_{CaT}, I_{leak} and I_{SOC}), the corresponding relative glucagon secretion rate (GS) and membrane voltage (V), which is mapped on the right y axis. (A) Low glucose conditions (G = 1 mM). (B) High glucose conditions (G = 6 mM).

Sensitivity Analysis

The stability analysis was performed for the two parameters, k_{direct} and $k_{indirect}$, which couple cAMP concentration with the electrophysiological model by [1]. As shown in Fig S2, k_{direct} is primarily responsible for the drop in RGS.

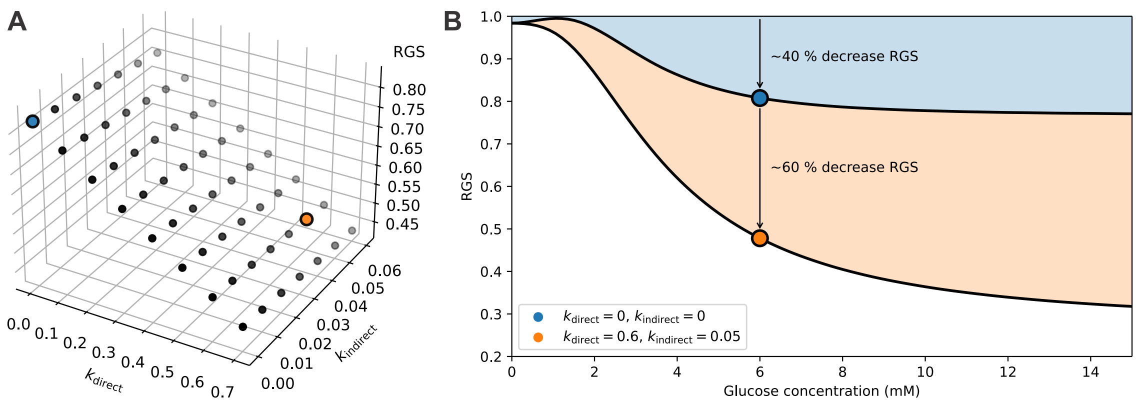

Fig S2. Sensitivity analysis of parameters k_{direct} and $k_{indirect}$. Blue dot in both panels represents RGS when both parameters are set to 0, and orange dot represents RGS with both parameter values as used in the model. (A) 3D plot of k_{direct}- and $k_{indirect}$-dependent RGS at the switching point (G = 6 mM). (B) Glucose-dependent RGS for the representative parameter values.

References

1. Montefusco, F.; Pedersen, M.G. Mathematical modelling of local calcium and regulated exocytosis during inhibition and stimulation of glucagon secretion from pancreatic alpha-cells. *J. Physiol.* **2015**, *593*, 4519–4530, doi:10.1113/JP270777.
